# Supplementary material for: A Bioengineering Approach for the Development of Fibroblast Growth Factor-7-Functionalized Sericin Biomaterial Applicable for the Cultivation of Keratinocytes
Source: Int J Mol Sci. 2022 Sep 1;23(17):9953. doi: 10.3390/ijms23179953 (PMC9456417; doi:10.3390/ijms23179953)
Supplement: Supplementary file 1 [file ijms-23-09953-s001.zip › ijms-1862489-supplementary.pdf]

Supplementary Materials

A Bioengineering Approach for Development of Fibroblast Growth Factor-7-functionalized Sericin Biomaterials Applicable for Cultivation of Keratinocytes

Ai Ai Lian[1, †], Yuka Yamaji[1, †], Kazuki Kajiwara[1], Keiko Takaki[1,2], Hajime Mori[1], Mervyn Wing On Liew[3], Eiji Kotani[1,2, *] and Rina Maruta[1*]

[1] Department of Applied Biology, Kyoto Institute of Technology, Sakyo-ku, Kyoto 606-8585, Japan

[2] Biomedical Research Center, Kyoto Institute of Technology, Sakyo-ku, Kyoto 606-8585, Japan

[3] Institute for Research in Molecular Medicine, Universiti Sains Malaysia, Minden, 11800, Penang, Malaysia

1. *Supplementary Results and Discussion*

Transgenic line S1sp-F7/poly producing cocoons containing human fibroblast growth factor-7 fused with the N-terminal H1-helix (polyhedron-encapsulation signal sequence; [1]) was generated and the inserted transgene was found flanked by sequences from chromosome 26 (Figure S1A (ii)) as determined by inverse PCR. However, sequences (shown as Ns in Figure S1A (ii)) at the right arm were not identified by the failure of the inverse PCR amplification due to unknown reasons.

The silkworm line S1sp-F7/poly × FH-P1A was generated to express recombinant H1/FGF-7 and polyhedrin as secreted proteins that will be incorporated into the sericin cocoon shells (Figure S1A (i)) after cocoon spinning. Our previous study showed that polyhedrin, expressed as a non-secreted protein in middle silk gland cells, is capable of crystallization [2]. Thus, we were prompted to further investigate whether the secreted polyhedrin can crystallize in the silk gland lumen and incorporated into cocoon shells following silk spinning. However, crystallized polyhedrin (also known as polyhedra) were

not observed in the sericin cocoon shells or MSG lumens, although the protein was detected in the sericin cocoon shells by western blot analysis (Figure S1B(ii)).

Altogether, this suggests that the crystallization process is not solely dependent on physiological environmental factors *in vivo*; but also the cellular mechanisms. Nonetheless, the sericin cocoon F7/poly-SC obtained from the S1sp-F7/poly × FH-P1A silkworm line showed similar biological activity and storage stability compared to the powder of F7-SC obtained from S1sp-F7 × FH-P1A (Figure S1 for the results generated using F7/poly-SC; see Figure 2, Figure 3, Figure 4, Figure 5, Figure 6, and Figure 7 for F7-SC powder in the main text). These results imply that the expression systems in MSG cells can support the production of multiple foreign proteins without compromising the productivity or bioactivity of the expressed proteins.

The slight difference in the H1/FGF-7 molecular mass observed between F7-PH crystals and F7/poly-SC (Figure S1B(i), Lane 1 and Lane 3) might be caused by the varied sample composition resulting from different treatments of polyhedra and sericin samples (main text section 4.5). In addition, the probable cleavage site of the sericin-1 promoter derived secretion signal peptide (consisting of the first 21 N-terminal amino acid residues) at the position 19 and 20 (Figure S2B) [3] also likely affected the H1/FGF-7 protein size detected in the F7/poly-SC sample because of the two amino acid residues remained. The *in vitro* crystallization of polyhedrin is challenging, and no successful attempts have been reported to date [2,4,5].

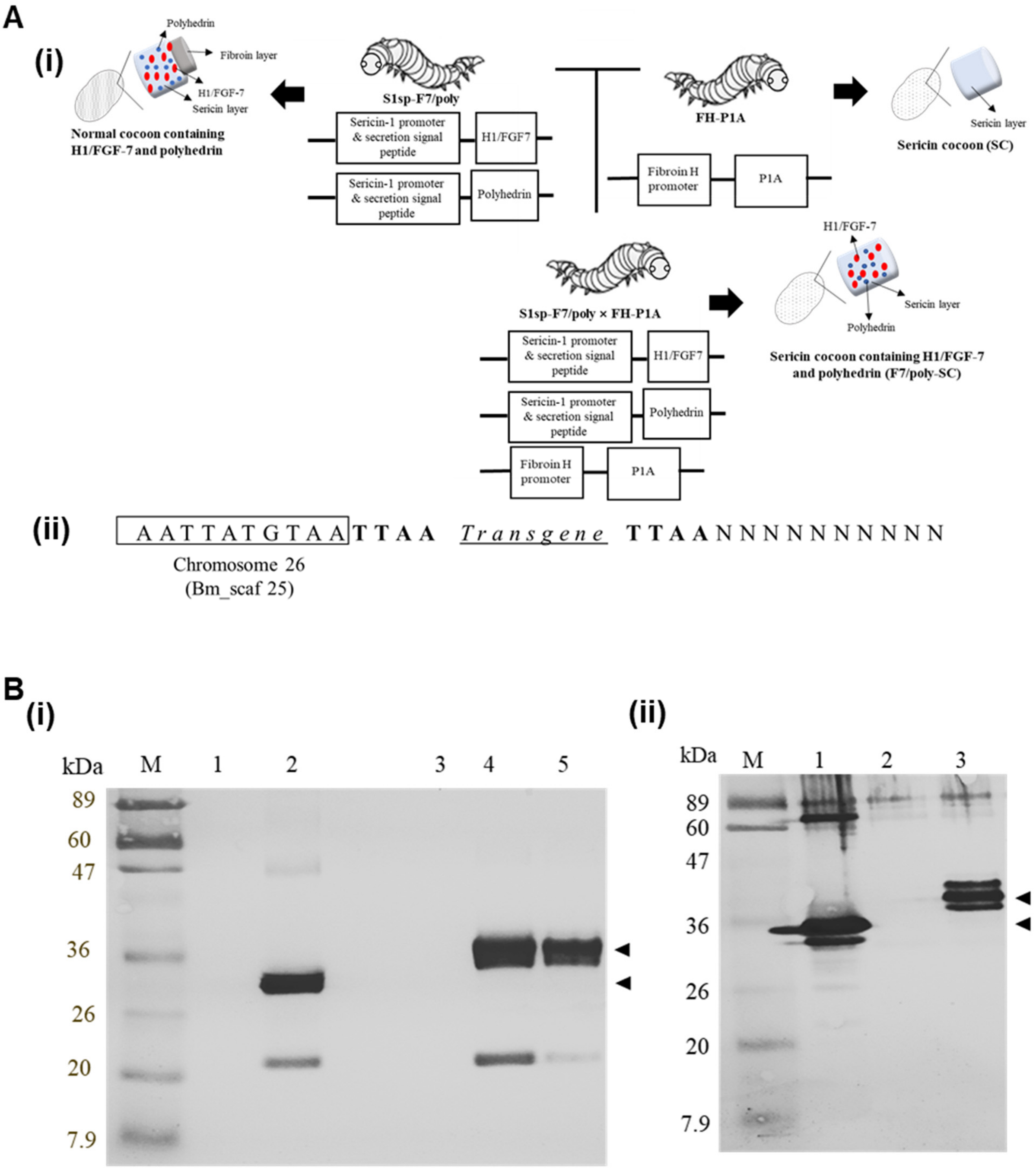
A
(i)
Polyhedrin
Fibroin layer
H1/FGF-7
Sericin layer
Normal cocoon containing H1/FGF-7 and polyhedrin
S1sp-F7/poly
Sericin-1 promoter & secretion signal peptide
H1/FGF7
Polyhedrin
FH-P1A
Fibroin H promoter
P1A
Sericin layer
Sericin cocoon (SC)
S1sp-F7/poly × FH-P1A
Sericin cocoon containing H1/FGF-7 and polyhedrin (F7/poly-SC)
(ii)
AATTATGTAA TTAA Transgene TTAA NNNNNNNNNN
Chromosome 26
(Bm_scaf 25)
B
(i)
kDa
M 1 2 3 4 5
89
60
47
36
26
20
7.9
(ii)
kDa
M 1 2 3
89
60
47
36
26
20
7.9

C

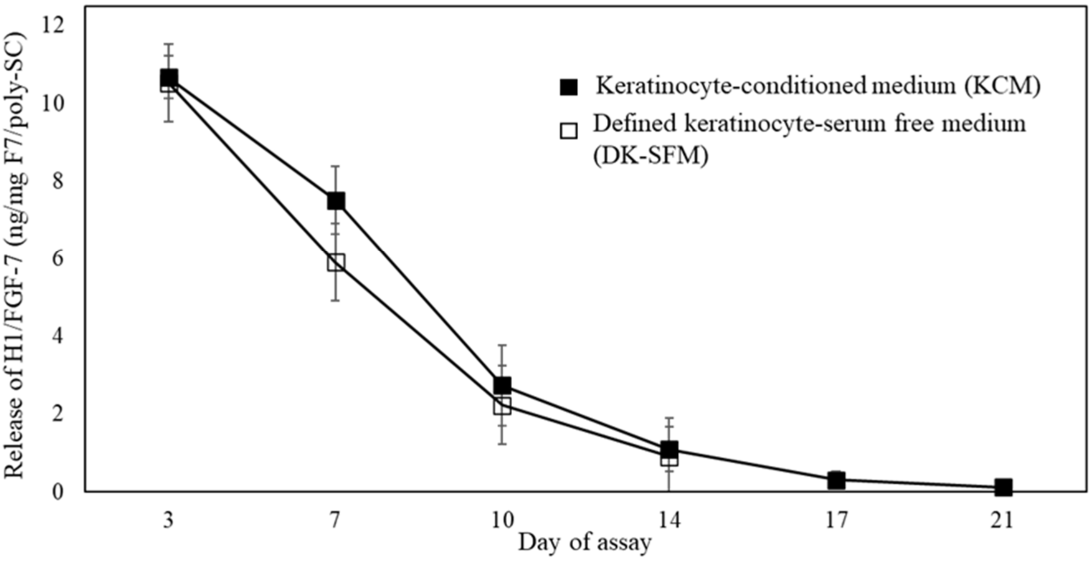
Release of H1/FGF-7 (ng/mg F7/poly-SC)
Keratinocyte-conditioned medium (KCM)
Defined keratinocyte-serum free medium (DK-SFM)
Day of assay
0
2
4
6
8
10
12
3
7
10
14
17
21

D

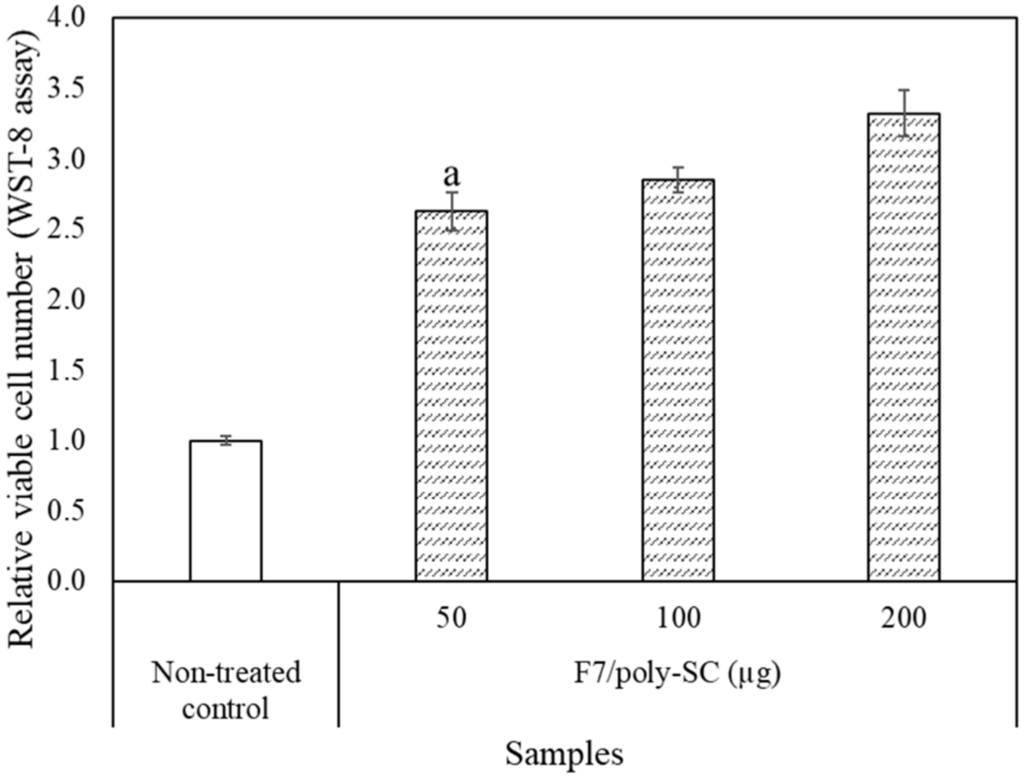
Relative viable cell number (WST-8 assay)
0.0
0.5
1.0
1.5
2.0
2.5
3.0
3.5
4.0
a
50
100
200
Non-treated control
F7/poly-SC (µg)
Samples

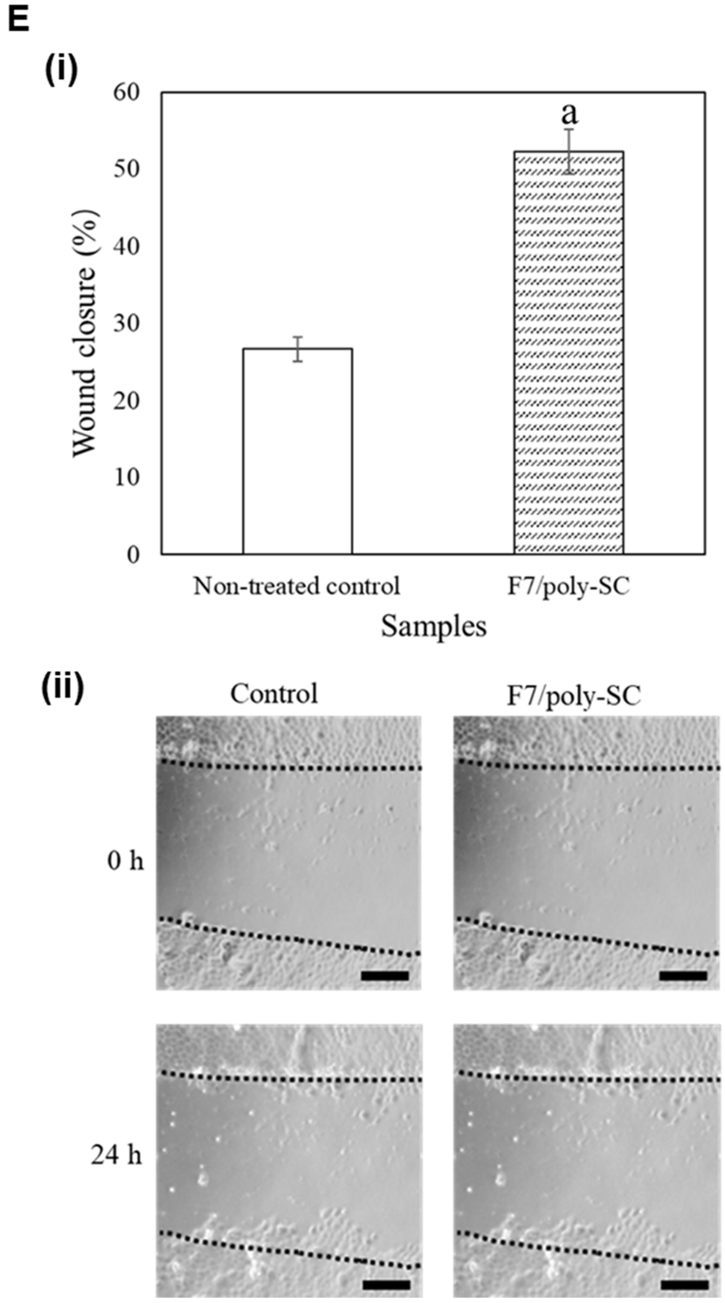
E
(i)
Wound closure (%)
60
50
40
30
20
10
0
a
Non-treated control
F7/poly-SC
Samples
(ii)
Control
F7/poly-SC
0 h
24 h

F

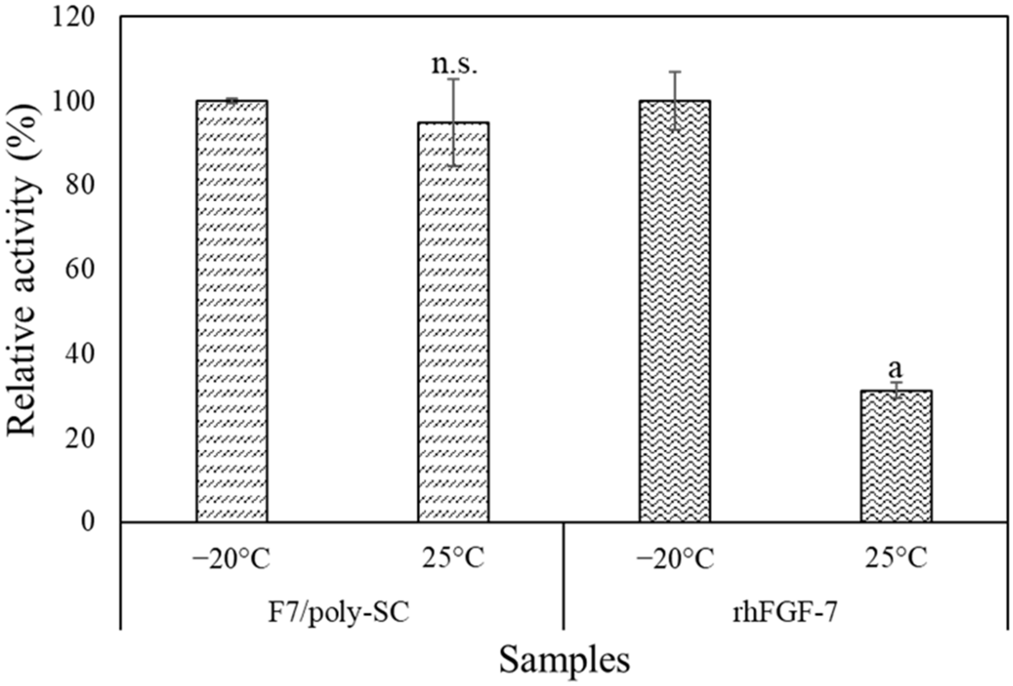

Relative activity (%)
120
100
80
60
40
20
0
n.s.
a
−20°C
25°C
−20°C
25°C
F7/poly-SC
rhFGF-7
Samples

G

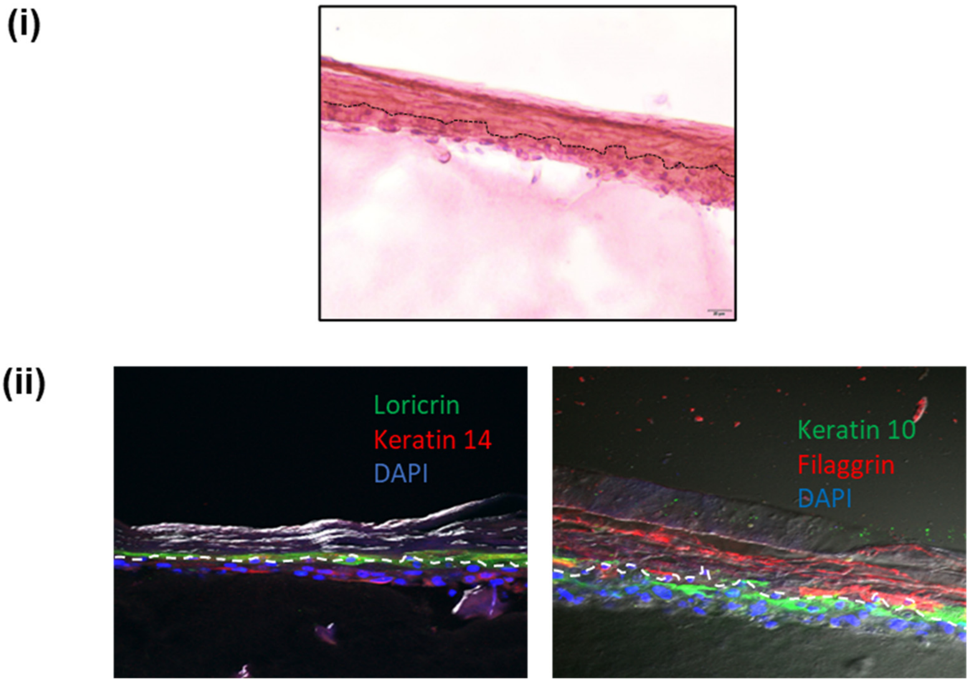

(i)
(ii)
Loricrin
Keratin 14
DAPI
Keratin 10
Filaggrin
DAPI

Figure S1. Generation of transgenic silkworm line S1sp-F7/poly × FH-P1A and the related experimental results. (**A**) Generation of the transgenic silkworms. (i) Transgenic silkworm lines and their corresponding cocoons. The transgenic line S1sp-F7/poly producing cocoons containing H1/FGF-7 and polyhedrin at the sericin layers was mated with the FH-P1A line that produces cocoons containing almost exclusively sericin (SC) to generate the S1sp-F7/poly × FH-P1A line. The resultant line S1sp-F7/poly × FH-P1A produces sericin cocoons incorporating H1/FGF-7 and polyhedrin (F7/poly-SC). (ii) Sequences from chromosome 26 of silkworms were found at the left arm of the inserted transgene in the line S1sp-F7/poly. The nucleotide sequences at the right arm could not be identified. (**B**) (i) Western blot analysis of H1/FGF-7. (Lane M) Protein molecular weight marker; 5×10^4 cubes of polyhedra obtained from Sf21 cell culture infected with (Lane 1, CPH) recombinant baculovirus carrying polyhedrin cDNA, and (Lane 2, F7-PH crystals) recombinant baculovirus carrying cDNA of H1/FGF-7 and polyhedrin [1]; 10 µg sericin cocoons derived from (Lane 3, SC) FH-P1A line, (Lane 4, F7-SC) S1sp-F7 × FH-P1A line, and (Lane 5, F7/poly-SC) S1sp-F7/poly × FH-P1A. Arrow heads indicate the position of H1/FGF-7. (ii) Western blot analysis of polyhedrin. (Lane M) Protein molecular weight marker; (Lane 1, CPH) 5×10^4 cubes of polyhedra obtained from Sf21 cell culture infected with recombinant baculovirus carrying polyhedrin cDNA; 10 µg sericin cocoons derived from (Lane 2, F7-SC) S1sp-F7 × FH-P1A line; and (Lane 3, F7/poly-SC) from S1sp-F7/poly × FH-P1A line. Arrow heads indicate the position of polyhedrin. (**C**) Release profile of H1/FGF-7 from F7/poly-SC powder suspended in aqueous media. The protein release profile examined using keratinocyte-conditioned medium (KCM, ■) and DK-SFM (□). (**D**) Proliferation of NHEK cells induced by F7/poly-SC powder. $^{a}p < 0.001$ vs. non-treated control. (**E**) Migration of NHEK cells induced by F7/poly-SC powder. $^{a}p < 0.001$ vs. non-treated control. (**F**) Short-term (7 days) storage stability of H1/FGF-7 incorporated into sericin cocoon powder containing polyhedrin. $^{n.s.}p > 0.05$ vs. -20°C counterpart and $^{a}p < 0.001$ vs. -20°C counterpart. (**G**) Three-dimensional (3D) cultivation NHEK cells on a collagen gel containing 800 µg F7/poly-SC powder. (i) Hematoxylin and eosin staining. (ii) Immunofluorescent staining. The parameters, experimental design, and data analysis were exactly the same as those described for F7-SC samples in the main text unless stated otherwise. Upper layer above the dashed line showed the speculated stratum corneum.

2. Supplementary Materials and Methods

2.1. Generation of pBacMCS [hr3-Ser1sp-H1/FGF-7, 3×P3-EGFP-SV40] Vector

The vector was constructed using primers listed in Table S1. The enhancer, hr3 sequence of the BmNPV-T strain [6] was PCR-amplified using primers 1 and 2 and inserted into the HindIII-BamHI site of the plasmid pIZ/V5-His (Invitrogen) to generate pIZ/V5-His

[hr3]. Sericin-1 promoter containing a secretion signal peptide sequence at the C-terminus (Ser1sp) [7], was amplified from *w1-pnd* strain using primers 3 and 4. pIZ/V5-His [hr3] was then digested with BamHI and XhoI to allow insertion of the Ser1sp to create pIZ/V5-His [hr3-Ser1sp]. The H1/FGF-7 cDNA was amplified with primers 5 and 6 and inserted into the XhoI-SacII sites downstream of the Ser1sp to obtain pIZ/V5-His [hr3-Ser1sp-H1/FGF-7].

The inserted sequence [hr3-Ser1sp-H1/FGF-7] together with the 3'-untranslated region of OpIE2 (downstream of pIZ/V5-His cloning sites), was amplified using primers 7 and 8. The obtained sequence was ligated into the BlnI-BlnI site of pBacMCS [3×P3-EGFP, UAS-SV40][8] using Ligation high Ver.2 (Toyobo Co., Ltd., Osaka, Japan). The final product pBacMCS [hr3-Ser1-H1/FGF-7, 3×P3-EGFP-SV40] is shown in Figure S2A (i).

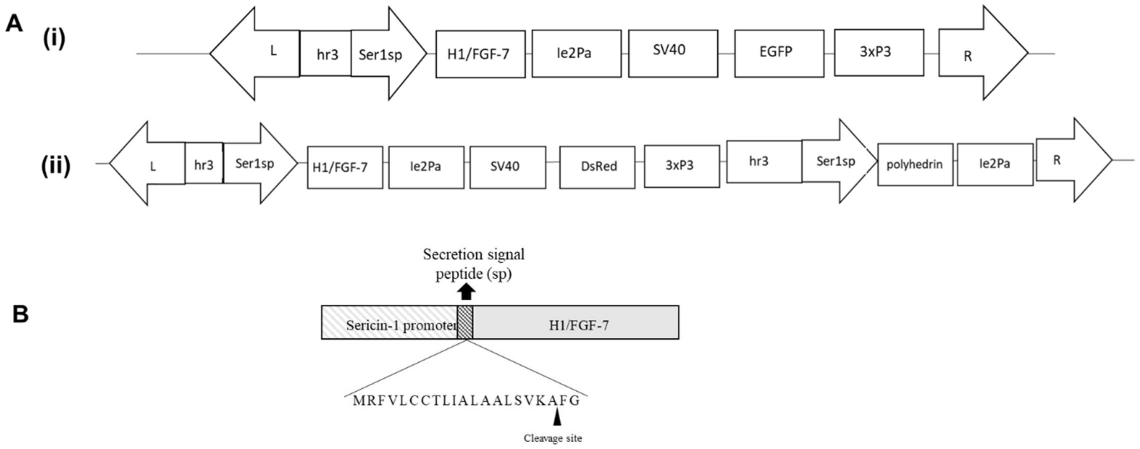

Figure S2. (**A**) Schematic diagram of the vectors used for germline transformation of *w1-pnd* silkworms. (i) The plasmid pBacMCS [hr3-Ser1sp-H1/FGF-7, 3×P3-EGFP-SV40] encoding H1-fused FGF-7 and marker EGFP was used to generate transgenic line S1sp-F7. (ii) The plasmid pBacMCS [hr3-Ser1sp-H1/FGF-7-SV40, 3×P3DsRed, hr3-Ser1sp-polyhedrin] encoding H1-fused FGF-7, polyhedrin, and DsRed was used to generate transgenic S1sp-F7/poly line. (**B**) Probable cleavage site of sericin-1 promoter-derived secretion signal peptide [3]. The box with the dark-grey slashes represents the secretion signal peptide (sp) and amino acid sequence is shown below. Arrow head indicates the cleavage site.

2.2. Generation of pBacMCS [hr3-Ser1sp-H1/FGF-7-SV40, 3×P3DsRed, hr3-Ser1sp-polyhedrin] Vector

The vector was constructed using primers listed in Table S1. The polyhedrin sequence [1] was amplified using primers 9 and 10 and cloned into the XhoI-SacII site of pIZ/V5-His [hr3-Ser1sp] to obtain pIZ/V5-His [hr3-Ser1sp-polyhedrin]. The [hr3-Ser1sp-polyhedrin], along with the 3'-untranslated region of OpIE2, was PCR-amplified using primers 11 and 12. The synthesized fragments [3×P3-DsRed-SV40][9] were amplified by PCR with primers 13 and 14. The [hr3-Ser1sp-H1/FGF-7] fragments were amplified using primers 15 and 16 from the aforementioned plasmid, pIZ/V5-His [hr3-Ser1sp-polyhedrin]. The three fragments obtained were cloned into BglII- and EcoRI-digested pBacMCS [3×P3-EGFP, UAS-SV40]. The final plasmid obtained, pBacMCS [hr3-Ser1sp-H1/FGF-7-SV40, 3×P3DsRed, hr3-Ser1sp-polyhedrin], is shown in Figure S2A (ii).

2.3. Generation of Transgenic Silkworm Line S1sp-F7/poly and S1sp-F7/poly × FH-P1A

The generation of transgenic silkworm line S1sp-F7/poly (Figure S1A) carrying genes for the expression of H1/FGF-7 and polyhedrin in MSGs was carried out by microinjection of pBacMCS [hr3-Ser1sp-H1/FGF-7-SV40, 3×P3-DsRed, hr3-Ser1sp-polyhedrin] and helper plasmid into the pre-blastodermic *w1-pnd* embryos. Screening for transgenesis was facilitated by the expression of the vector-derived DsRed. S1sp-F7/poly was mated with FH-P1A and the progenies were sib-mated to obtain homogeneous line S1sp-F7/poly × FH-P1A producing sericin cocoons that incorporated with H1/FGF-7 and polyhedrin.

2.4. Inverse PCR Analysis of Transgenic Silkworms, S1sp-F7 and S1sp-F7/poly

The genomic DNA obtained from the G2 generation of the transgenic lines was used for inverse PCR analysis. Silk glands were first collected from fifth instar larvae and incubated overnight with proteinase K prior to genomic DNA purification using the standard phenol treatment method. Purified genomic DNA was digested with Sau3AI and ligated overnight at 16°C for circularization using Ligation high Ver. 2 (Toyobo Co., Ltd., Osaka, Japan). The circularized sample was used as the template for inverse PCR amplification. The fragments in the vicinity of the *piggyBac* derived-inverted terminal repeats were amplified using primers 17–20 (Table S1). The PCR amplified products were then analyzed by DNA sequencing.

2.5. Experimental Design and Parameters Related to the Analysis of F7/poly-SC.

2.5.1. Preparation of Sericin Cocoon Powders

The sericin cocoons collected from transgenic line S1sp-F7/poly × FH-P1A were pulverized for 10 s using a blender (Wonder Blender Osaka Chemical Co., Ltd., Osaka, Japan), lyophilized, and kept at -20°C until further use.

2.5.2. Western Blot Analysis of Polyhedrin and H1/FGF-7

The presence of H1/FGF-7 and polyhedrin in the sericin cocoons obtained from S1-F7/poly × FH-P1A (F7/poly-SC) was examined by western blotting. Dissolved F7/poly-SC (10 μg), 5×10^4 cubes of cypovirus polyhedra encapsulating H1/FGF-7 (F7-PH crystals, [1]) and 5×10^4 cubes of cypovirus polyhedra encapsulating no foreign protein (CPH) were used

for for the analysis. Polyhedrin was probed with the rabbit antiserum and followed by incubation with goat-anti rabbit IgG conjugated with horseradish peroxidase [4].

2.5.3. *Release Profile of H1/FGF-7 from F7/poly-SC Powder*

Examination on H1/FGF-7 released from F7/poly-SC powder (0.04%, w/v) into keratinocyte-conditioned medium (KCM) and basal medium DK-SFM was performed exactly the same as that described for F7-SC powder in the main text (section 4.6)

2.5.4. P*roliferation and Migration of Normal Human Epidermal Keratinocyte (NHEK) Cells in the Presence of F7/poly-SC Powder*

Proliferation of NHEK cells with different doses (50, 100 and 200 μg) of F7/poly-SC powder and migration of serum-starved NHEK cells with 200 μg F7/poly-SC powder was performed exactly the same as that described for F7-SC powder in the main text (section 4.7 for proliferation of NHEK cells and section 4.8 for migration of NHEK cells).

2.5.5. *Storage Stability of H1/FGF-7 Incorporated into Sericin Cocoon Powder of S1sp-F7/poly × FH-P1A Line*

Sericin cocoons F7/poly-SC suspension (2 mg/mL) in phosphate buffered-saline was used to analyze bioactivity of the incorporated H1/FGF-7 on proliferation of NHEK cells after being stored at 25°C or -20°C for 1 week. The analysis was performed exactly the same as that described for F7-SC powder in the main text (section 4.9).

2.5.6. *Three-dimensional (3D) Cultivation of NHEK Cells using Collagen Gels Containing F7/poly-SC Powder*

Powder of F7/poly-SC powder (800 μg) was added to the collagen for 3D cultivation of NHEK cells. The cultivation of NHEK cells, and analysis of the 3D-cultured model including hematoxylin and eosin staining, and immunohistochemical staining were performed as that described in section 4.10 of the main text.

Table S1. The oligonucleotide primers used for vector construction (1–16) and inverse PCR analysis of the silkworm transgenesis (17–20). The restriction sites are indicated in bold.

No.	Name	Sequence
1	hr3_5'	TTT**AAGCTT**AAAAAGAAGCCGTGCCCA
2	hr3_3'	TTT**GGATCC**CAGCGTCGTGAAAAGAGG
3	Ser1_5'	TTT**GGATCC**AGCGGTCAGAAACCTTG
4	Ser1_3'	TTT**CTCGAG**ACCGAAAGCTTTTACGCTGGAGCGCAGCCAA
5	H1/FGF-7_5'	TTT**CTCGAG**ATGGCAGACGTAGCAG
6	H1/FGF-7_3'	TTT**CCGCGG**TTAAGTTATTGCCATAGG
7	hr3(pIZ to pBAc)	TTT**CCTAGG**AAAAAGAAGCCGTGCCCA
8	OpIE2(pIZ to pBAc)	TTT**CCTAGG**CCACGCGCTTGAAAGGAGTGTG
9	IZ-poly F	TTT**CTCGAG**ATGGCAGACGTAGCAGGAAC
10	pIZ-poly R	TTT**CCGCGG**CTACTGACGGTTACTCAGAG
11	Poly F	AACATTGTC**AGATCT**AAAAGAAGCCGTGCCCAGCCAC
12	Poly R	GGCGAGCTCGAATTATCCCCTGATTCTGTGGATACCG
13	DsRed F	TAATTCGAGCTCGCCCGGGG
14	DsRed R	AGATCGTTGCGGGCTGAGTTTGGACAAACCACAACTAG
15	FGF-7 F	AGCCCGCAACGATCTGGTAAAC
16	FGF-7 R	CGCGCCATC**GAATTC**CACGCGCTTGAAAGGAGTGTG
17	*piggyBac* right ITR 1: 5'	CGCATGATTATCTTTAACGTACGTCAC
18	*piggyBac* right ITR 2: 3'	GGGGTCCGTCAAAACAAAACATC

19	*piggyBac* left ITR 1: 5’	GAGTCTCTGCACTGAACATTGTCA
20	*piggyBac* right ITR 2: 3’	ATCAGTGACACTTACCGCATTGACA

Supplementary References:

1. Ijiri, H.; Coulibaly, F.; Nishimura, G.; Nakai, D.; Chiu, E.; Takenaka, C.; Ikeda, K.; Nakazawa, H.; Hamada, N.; Kotani, E.; Metcalf, P.; Kawamata, S.; Mori, H. Structure-based targeting of bioactive proteins into cypovirus polyhedra and application to immobilized cytokines for mammalian cell culture. *Biomaterials* **2009**, *30*, 4297–4308. doi: https://doi.org/10.1016/j.biomaterials.2009.04.046

2. Maruta, R.; Takaki, K.; Yamaji, Y.; Sezutsu, H.; Mori, H.; Kotani, E. Effects of transgenic silk materials that incorporate FGF-7 protein microcrystals on the proliferation and differentiation of human keratinocytes. *FASEB BioAdv.* **2020**, *2*, 734–744. doi: https://doi.org/10.1096/fba.2020-00078

3. Guo, X.; Zhang, Y.; Zhang, X.; Wang, S.; Lu, C. Recognition of signal peptide by protein translocation machinery in middle silk gland of silkworm *Bombyx mori*. *Acta Biochim. et Biophys. Sin.* **2008**, *40*, 38–46. doi: https://doi.org/10.1111/j.1745-7270.2008.00376.x

4. Kotani, E.; Yamamoto, N.; Kobayashi, I.; Uchino, K.; Muto, S.; Ijiri, H.; Shimabukuro, J.; Tamura, T.; Sezutsu, H.; Mori, H. Cell proliferation by silk gut incorporating FGF-2 protein microcrystals. *Sci. Rep.* **2015**, *5*, 1-10. doi: https://doi.org/10.1038/srep11051

5. Chiu, E.; Coulibaly, F.; Metcalf, P. Insect virus polyhedra, infectious protein crystals that contain virus particles. *Curr. Opin. Struc. Biol.* **2012**, *22*, 234–240. doi: https://doi.org/10.1016/j.sbi.2012.02.003

6. Wang, F.; Xu, H.; Yuan, L.; Ma, S.; Wang, Y.; Duan, X.; Duan, J.; Xiang, Z.; Xia, Q. An optimized sericin-1 expression system for mass-producing recombinant proteins in the middle silk glands of transgenic silkworms. *Transgenic Res.* **2013**, *22*, 925–938. doi: https://doi.org/10.1007/s11248-013-9695-6

7. Tatematsu, K.I.; Kobayashi, I.; Uchino, K.; Sezutsu, H.; Iizuka, T.; Yonemura, N.; Tamura, T. Construction of a binary transgenic gene expression system for recombinant protein production in the middle silk gland of the silkworm *Bombyx mori*. *Transgenic Res.* **2010**, *19*, 473–487. doi: https://doi.org/10.1007/s11248-009-9328-2

8. Sakudoh, T.; Sezutsu, H.; Nakashima, T.; Kobayashi, I.; Fujimoto, H.; Uchino, K.; Banno, Y.; Iwano, H.; Maekawa, H.; Tamura, T.; Kataoka, H.; Tsuchida, K. Carotenoid silk coloration is controlled by a carotenoid-binding protein, a product of the yellow blood gene. *Proc. Natl. Acad. Sci. U.S.A.* **2007**, *104*, 8941-8946. doi: https://doi.org/10.1073/pnas.0702860104

9. Yonemura, N.; Tamura, T.; Uchino, K.; Kobayashi, I.; Tatematsu, K.; Iizuka, T.; Sezutsu, H.; Muthulakshmi, M.; Nagaraju, J.; Kusakabe, T. PhiC31 integrase-mediated cassette exchange in silkworm embryos. *Mol. Genet. Genomics* **2012**, *287*, 731–739. doi: https://doi.org/10.1007/s00438-012-0711-y
